# Impact of Anthropomorphic Shape and Skin Stratification on Absorbed Power Density in mmWaves Exposure Scenarios

**DOI:** 10.3390/s25144461

**Published:** 2025-07-17

**Authors:** Silvia Gallucci, Martina Benini, Marta Bonato, Valentina Galletta, Emma Chiaramello, Serena Fiocchi, Gabriella Tognola, Marta Parazzini

**Affiliations:** 1 Istituto di Elettronica e di Ingegneria dell’Informazione e delle Telecomunicazioni (IEIIT), Consiglio Nazionale delle Ricerche (CNR), 20133 Milan, Italy; 2 Department of Electronics, Information and Bioengineering (DEIB), Politecnico di Milano, 20133 Milan, Italy

**Keywords:** absorbed power density, computational dosimetry, mmWave wearable device, multi-layer models, plane wave exposure, realistic human models

## Abstract

As data exchange demands increase also in widespread wearable technologies, transitioning to higher bandwidths and mmWave frequencies (30–300 GHz) is essential. This shift raises concerns about RF exposure. At such high frequencies, the most crucial human tissue for RF power absorption is the skin, since EMF penetration is superficial. It becomes thus very important to assess how the model used to represent the skin in numerical dosimetry studies affects the estimated level of absorbed power. The present study, for the first time, assesses the absorbed power density (APD) using FDTD simulations on two realistic human models in which: (i) the skin has a two-layer structure made of the stratum corneum and the viable epidermis and dermis layers, and (ii) the skin is modelled as a homogeneous dermis stratum. These results were compared with ones using flat phantom models, with and without the stratified skin. The exposure assessment study was performed with two sources (a wearable patch antenna and a plane wave) tuned to 28 GHz. For the wearable antenna, the results evidence that the exposure levels obtained when using the homogeneous version of the models are always lower than the levels in the stratified skin version with percentage differences from 16% to 30%. This trend is more noticeable with the female model. In the case of plane wave exposure, these differences were less pronounced and lower than 11%.

## 1. Introduction

In recent years, we have been witnessing the global proliferation of the “smart world,” the concept that interconnected smart environments enhance efficiency, connectivity, and automation across various sectors, including healthcare, transportation, and urban infrastructure [1]. Wearable devices, defined in IEEE 802.15.6 [2] as constitutive elements of wireless body area networks (WBANs), use communication protocols such as Bluetooth, ZigBee, and Bluetooth Low-Energy (BLE) at 2.45 GHz [3]. These devices transmit data wirelessly to external gateways. As data exchange demand is constantly growing, enhancing bandwidth and communication frequency to the millimeter-wave spectrum (30–300 GHz) becomes essential. Fifth-generation NR is the technology that allows to utilize millimeter-wave bands for wireless communication, in particular for the implementation of “smart world” [4].

The advent of such smart wearable and interconnected devices potentially impacts the level of exposure to electromagnetic fields because these devices are worn in contact with the body or, at most, at a distance equal to the minimum thickness of the clothing. Moreover, the use of novel mmWave bands changes the exposure scenario in which people are immersed and, in this regard, the key factors to be considered comprise, for example, the shape of the main beam, the frequency of the EMF, etc. Indeed, the use of mmWaves practically means that the power emitted by these smart interconnected devices is absorbed by the human body mainly at superficial tissues, i.e., the skin, given the inverse relationship between frequency (f) and EMF penetration depth (δ). This implies that, at such high frequencies, the skin becomes the primary tissue affected by EMF absorption and the modelling of this tissue is one of the issues that is still open in electromagnetic dosimetry above 6 GHz [5].

From the point of view of EMF absorption, two skin layers play a significant role, namely the stratum corneum (SC) and the viable epidermis and dermis. As these two layers have different dielectric properties (Table 1) [6], this could generate reflection phenomena that might impact the way in which the power of the RF EMF is absorbed by the skin. Thus, in case of EMF exposure assessment, the skin model plays a crucial role in making assessments about the interaction between electromagnetic fields and the human body accurate, especially when working at mmWaves. Also, the thickness of the skin, and in particular the thickness of the SC, is not uniform across the whole body but varies with body region: on the palms and soles it ranges at around 0.02–0.7 mm (thick SC), whereas on the rest of the body it becomes thinner, ranging from 0.01 mm to 0.02 mm (thin SC) [7].

However, from the regulatory point of view, there is no rigorous definition regarding the skin layer at which to calculate the absorbed power density (APD, W/m^2^), and this reflects uncertainty about the skin model to be used in dosimetric studies. Indeed, the International Commission on Non-Ionizing Radiation Protection (ICNIRP) in its Guidelines [8] did not specify the skin model to be employed nor, consequently, the skin layer for which to evaluate the APD, which is defined as the parameter of interest for frequencies > 6 GHz. On the other hand, the Institute of Electrical and Electronics Engineers (IEEE) in its IEEE C95.1-2019 [9] mentioned the “epithelial power density” as the parameter of interest for frequencies > 6 GHz, referring to the stratum corneum as the layer to be considered. This uncertainty linked to the virtual model to be used in computational dosimetry is symptomatic of the need to develop reliable models to investigate and analyze in research areas such as product safety and human protection, which are of interest to electromagnetic compatibility (EMC) [10]. In the literature, there are several studies that investigated how skin modelling affects the EMF dose of exposure. Christ et al. [6] aimed to study the variation in temperature increase using a layered skin structure. The study involved a plane wave (S_inc_ = 10 W/m^2^) at frequencies ranging from 6 to 100 GHz, impinging on a flat four-layer model of the most external human tissues. The stratum corneum (SC) was modelled as both a thin (i.e., 0.01 mm or 0.02 mm depending on the frequency) and thick layer (i.e., 0.02 mm or 0.7 mm). The study found that modelling the skin as a homogeneous layer led to an underestimation of the temperature increase compared to the multi-layer structure. In a follow-up study [11], the same group of authors addressed the calculation of the power transmission coefficient with the same models of the previous study but also by varying the incidence angle of the impinging plane wave tuned to 6 GHz, 30 GHz, 60 GHz, and 300 GHz, with different polarizations. They found that the power transmission coefficient for the perpendicular component of the Poynting vector is affected by the stratification of the skin; specifically, modelling a thick SC, the power transmission coefficient was consistently higher than for the homogeneous layer model.

Furthermore, Alekseev et al. [12] studied the reflection characteristics with four different flat models, ranging from a homogeneous dermis to a four-layer structure. The study focused on the forearm model made of thin-layer skin (i.e., 0.015 mm) and a palm model made of thick-layer skin (i.e., 0.42–0.43 mm). The results revealed that, in the range of 37–74 GHz, the thin SC did not significantly affect the reflection of millimeter waves (mmWaves), whereas the thick SC behaved as a matching layer, which caused a reduction in the reflection data. The same authors extended a previous study [13] by estimating the specific absorption rate (SAR), penetration depth, and power density in the same forearm and palm models with plane waves in the range of 30–300 GHz. This latter study confirmed that the thin SC had minimal influence on the interactions between mmWaves and skin.

Sasaki et al. [14] focused their study on the interaction between a plane wave and a flat multi-layer model in which the SC was not included: the skin model consisted of the epidermis and dermis strata of different thicknesses. In a frequency range from 10 GHz to 1 THz, by comparing the skin thickness of the forearm, triceps, quadriceps, and abdomen, the authors of [14] demonstrated that the transmittance and temperature elevation were affected by the thickness of the skin as a whole, without specifically addressing the SC layer.

Finally, Gallucci et al. [15] estimated exposure levels in four different flat multi-layer models under two distinct exposure conditions: a wearable antenna and a plane wave, both operating at 28 GHz and 39 GHz. The skin was modelled as a two-layer structure with the first layer consisting of thin SC (i.e., 0.02 mm) and the innermost layer comprising a viable epidermis and dermis. The results revealed that the impact of the SC on exposure levels was more evident in exposures due to wearable antennas, whereas for exposure induced by the plane wave, the impact of skin thickness was less significant, and the differences compared to the homogeneous model were minimal. Although it is clear from this brief overview that the literature lacks a common consensus on the approach to be used for modelling the skin when studying its interaction with mmWaves, most of the cited works revealed that the introduction of skin stratification in the studied model provided different findings regarding the exposure dose, when compared with the single homogeneous layer model. These results were obtained from both computational and analytical studies, also showing how the computational and analytical methods are congruent with each other. However, all these studies share the common feature of employing a flat model where the human body is modelled as a planar phantom, so neglecting the actual shape of human bodies.

In this regard, there are studies affirming that the shape of anatomical districts affects the EMF power absorption; indeed, when an irregular skin model is employed, its shape influences the EMF energy absorption [16]. Colella et al. [17] investigated the power absorbed when using the common flat model and an adult virtual human model (‘Duke’ from the Virtual Population (ViP) [18]), in three anatomical districts when exposed to mmWave frequencies. Both in the flat and in the anatomical model, the skin was modelled as a homogeneous layer. The paper showed that the differences in the exposure estimated with these two modelling approaches are non-negligible. Moreover, Sacco et al. [19] and Kapetanovic et al. [20] demonstrated that the absorbed power density estimated in realistic anatomical regions varies depending on the ratio between the radius of the curvature of the irradiated region and the wavelength of the field. Likewise, Diao et al. [21] revealed that the averaged absorbed power density (APD) and the temperature rise in body models with non-planar surfaces were influenced by frequency and curvature, for electromagnetic field exposure above 6 GHz.

According to the abovementioned literature, the irregularities of human body geometry alter exposure levels, as the surface exposed to EMF is non-uniform, leading to variations in the interaction with the electromagnetic field. However, accounting for these irregularities presents computational challenges. Since exposure metrics are averaged over volumes or surfaces, this process compresses point-specific values into single approximations [20], which become increasingly coarse with geometric discontinuities. Studies consistently demonstrate that anatomical irregularities affect exposure assessment results, revealing limitations in planar tissue modelling. This effect becomes particularly pronounced at high frequencies where the RF wavelength approaches the dimensions of anatomical curvatures and irregularities, necessitating realistic human models for accurate exposure assessment.

So far, the integration of the inner structure of the skin and the anthropomorphic shape of realistic human bodies has been considered costly and hard to implement [22], so much so that it has never been addressed. As illustrated in Table 2, the multi-layer skin structure and anthropomorphic shape have never been addressed in the same model in any previous study.

Instead, the present work aims to fill this gap, merging the realistic anthropomorphic shape of the human body and the inner structure of the skin by implementing for the first time a multi-layer skin model in virtual human models that retain the irregularities in the geometry of real human bodies. For the sake of completeness, this work assessed the dose absorbed at 28 GHz under two different exposure conditions: (i) exposure due to a wearable patch antenna, and (ii) exposure due to a plane wave. In both cases, the human body was modelled as geometrically realistic and with the inner stratification of the skin implemented, with particular focus on two anatomical regions related to the typical way wearable devices are used—the trunk and the wrist.

One of the innovative aspects of the present work is therefore the use of a two-layer skin model, in anatomically realistic models that comprise a 0.02 mm SC layer. The same exposure conditions were also applied to a homogeneous (dermis) and to four-layer (SC, dermis, fat, and muscle) flat models in order to comprehend the impact on the exposure levels due to a shape typical of the human body. Evaluation of the effect on the absorbed power density of the irregularities of actual human bodies due to their anthropomorphic shape and the effect of the skin stratification was performed by means of a computational approach that utilized the finite-difference time-domain (FDTD) method.

## 2. Materials and Methods

The current section describes the characteristics of the simulated scenarios, specifically the human models (Section 2.1) and the RF-EMF sources (Section 2.2). Section 2.3 provides information regarding the numerical approach used to assess exposure levels.

### 2.1. Model Characteristics

**Figure 1:**
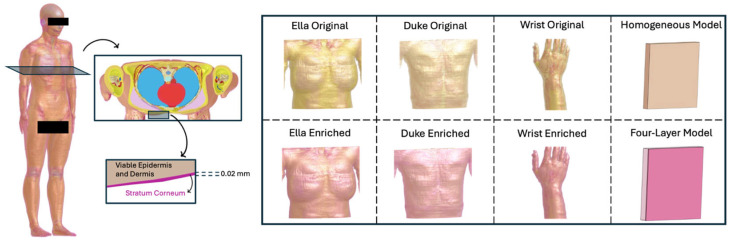
The ‘enriched geometry’ implemented in our study. On the **left**, the procedure to enrich the anatomically realistic model; as an example, the Ella model is reported. On the **right**, all the used models, in their Original and Enriched versions, are reported.

In the present study, simulations were performed on the anatomical models of an adult female and male model, respectively, Ella (age = 26 yo., height = 1.63 m, weight = 57.3 kg, BMI = 21.5 kg/m^3^) and Duke (age = 34 yo., height = 1.77 m, weight = 70.3 kg, BMI = 22.4 kg/m^3^) taken from the Virtual Population [18]. In these models the skin is modelled as a single layer, i.e., a viable epidermis and dermis. In the present study we aimed to model the skin as two layers comprising the SC (the outermost layer) and the viable epidermis and dermis (the inner layer). For this purpose, the two adult models were modified in this study by introducing a layer of SC of 0.02 mm thickness over the whole body, following its shape. Indeed, this SC thickness is valid for almost the entire body surface, except for some specific locations (such as the palms and soles) [6] that are not of interest for exposure due to wearable devices. For clarity purposes, henceforth, the two models lacking the SC will be designated as Duke/Ella Original, while the other two will be termed Duke/Ella Enriched. Figure 1 illustrates the approach used to implement the SC in the realistic human models and the Original and Enriched versions used in our study. The dielectric properties of the stratum corneum and the viable epidermis and dermis at 28 GHz for both types of human models were taken from the literature [7] and are reported in Table 1, while the dielectric properties at 28 GHz of all the other tissues of the human models were set according to data from the literature [23].

**Figure 2:**
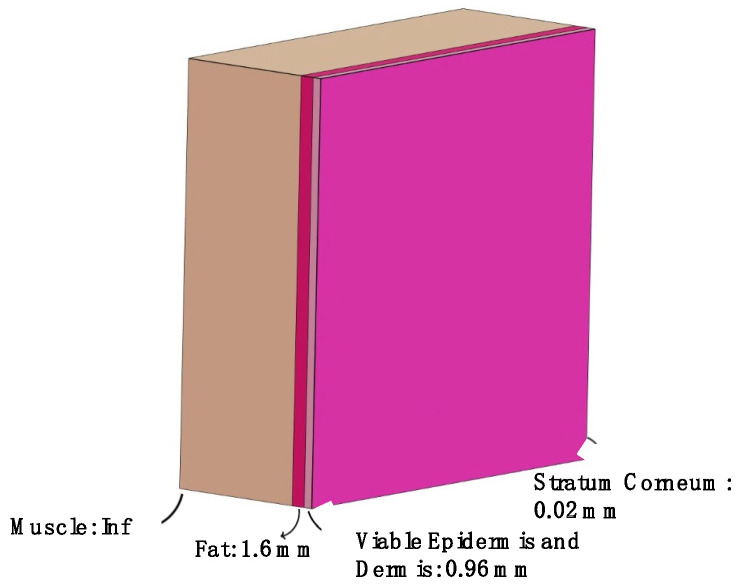
The four-layer model with the details of the thicknesses of each layer. For the sake of readability, the width and length of the model are scaled differently from the thickness.

Furthermore, in order to highlight the effect of an anthropomorphic shape on the EMF dose results, two flat models (see Figure 1 and Figure 2) were also modelled, consisting of a homogeneous flat model made of only the viable epidermis and dermis, and a four-layer flat model made by, from the outermost to the innermost layer, the SC, a viable epidermis and dermis, fat, and muscle, thereby mimicking the external layers of the body of the enriched version of virtual human bodies. The choice to also include the fat and muscle was made to simulate a flat phantom that is as similar to the virtual human model as possible. All these tissues were characterized through their dielectric properties assigned according to the literature, [[6] see also Table 1]. The overall dimensions of both flat models were 150 × 150 × 150 mm^3^. For the four-layer model, the thickness of each layer was derived from the literature [15], averaging the thicknesses according to those of the tissues in both the simulated anatomical districts.

### 2.2. RF-EMF Sources and Exposure Scenarios

The numerical human and flat models used in the present work were exposed to two different RF sources, one at a time: a wearable patch antenna and a plane wave, both at 28 GHz.

The model with the wearable patch antenna was derived from Chahat et al. [24]. It consisted of an antenna for off-body communications modelled as a microstrip-fed four-patch array antenna. The original antenna was re-tuned in free space [15] in order to emit at 28 GHz (S_11_ @ 28 GHz = −13.5 dB). The ground and the patch of the antenna were modelled as copper (ε_r_ = 1, σ = 5.813·10^7^ S/m), whereas the substrate was modelled as RT Rogers Duroid 5880 dielectric (ε_r_ = 2.2, σ = 5·10^−4^ S/m), with an overall size of 40 × 16.62 mm and inter-element array distance of 9.6 mm.

**Figure 3:**
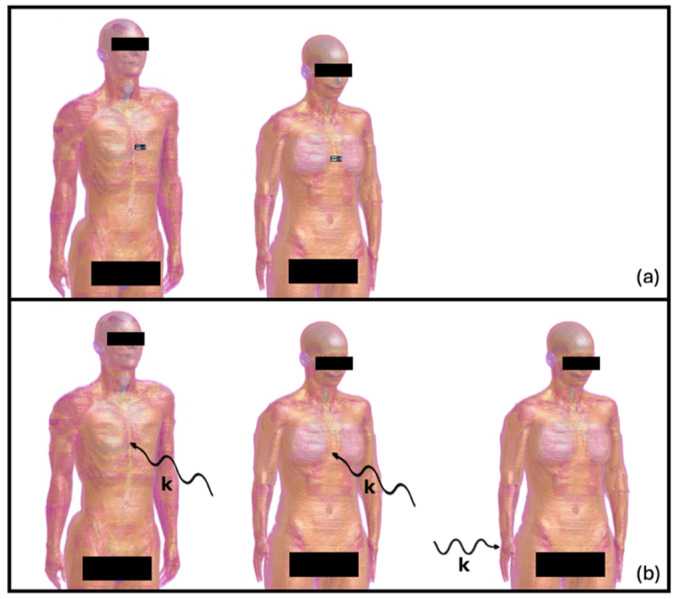
The exposure scenarios in which the realistic virtual human models are used: in panel (**a**), from left to right, the Duke and Ella models with the wearable antenna placed on their trunk; in panel (**b**), from left to right, the Duke trunk, Ella trunk, and Ella wrist scenarios with the incidence of the TEM-polarized plane wave.

The patch antenna was placed at a distance that took into account the minimum thickness of clothing, i.e., 2 mm, from the skin of the human models, at the level of the trunk, in order to mimic the realistic use of wearable antennas for off-body communication. For consistency, the same portion of the trunk was also exposed to a TEM-polarized plane wave, whose direction of propagation was set as perpendicular to the skin of the human model. Moreover, to mimic another actual usage of a wearable device (e.g., a smartwatch), the wrist of Ella was impinged by a plane wave TEM-polarized with perpendicular incidence with respect to the surface of the wrist region itself to simulate the worst-case exposure scenario [15]. Figure 3 shows the simulated scenarios in which the virtual human models are involved.

Concerning the flat human models, for each of them the patch antenna was positioned at the centre of the flat model and located 2 mm from it. Moreover, each flat model was also irradiated by a TEM-polarized plane wave with a perpendicular incidence.

### 2.3. Exposure Assessment

EMF exposure was assessed with the finite-difference time-domain (FDTD) method, as implemented in the electromagnetic simulation software Sim4Life v.8.0 (ZMT Zurich Med Tech AG, Zurich Switzerland, www.zmt.swiss (accessed on 15 July 2025)). Concisely, the FDTD method involves both a spatial and temporal discretization of the electric and magnetic fields over a period of time and a specific spatial domain limited with boundary conditions.

The computational domain, including the EMF source (i.e., the wearable antenna and the plane wave) and the human/flat model, was discretized with a non-uniform grid with a mesh cell size varying from 0.001 mm to 0.32 mm, depending on the investigated exposure scenario. The grid size of the models was maintained in all the simulations in order to guarantee consistency in the comparison of the results.

Both human models were truncated in the simulations to focus exclusively on the body region where the EMF source was positioned, i.e., the trunk or the wrist. The boundary conditions were set as absorbing conditions with the Perfect Matched Layer (PML). Even for the flat models, all the boundary conditions were set as PML. The simulations were performed on a Z8 16-Core Processor @ 3.8 GHz, RAM 512 GB workstation with an NVIDIA GeForce RTX5000 graphics card. To speed up the simulations, the Sim4Life GPU accelerator aXware was also used.

For exposure assessment, we calculated the absorbed power density (APD, W/m^2^) and its spatial peak (psAPD); these were the quantities used to assess the exposure to EMF in the range 6–300 GHz [8]. Since the ICNIRP Guidelines suggest averaging the APD over 1 cm^2^ surface area instead of 4 cm^2^, for frequencies greater than 30 GHz, we used the smallest area to account for focal beam exposure, thereby performing a conservative study. The size of the averaging area, i.e., 1 cm^2^, was derived from the edge of the averaging volume of the specific absorption rate (SAR) of 1 g [25]. In more detail, the APD (1) was calculated by integrating the real part of the Poynting vector ($E\times H^{*}$) over a square area (A), using a rotating square as the planar averaging surface.

(1) $$\mathrm{APD}=\frac{1}{A}\iint_{A}Re\left[E\times H^{*}\right]dS$$

To characterize the spatial distribution of the APD, we also calculated the percentages of APD values greater than 90% of the psAPD, the percentage between 90% and 70% of the psAPD, and the percentage of data below 70% of the psAPD.

## 3. Results

**Figure 4:**
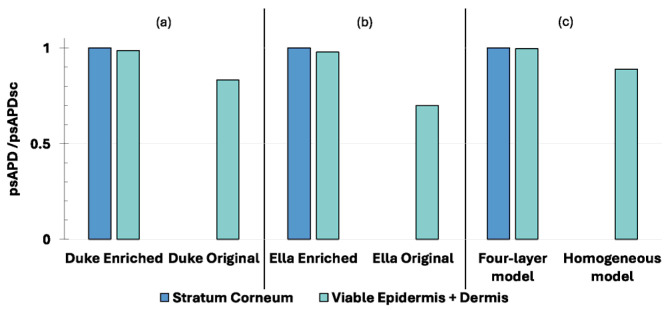
The normalized peaks of the APD averaged over 1 cm^2^ of skin tissue when the source is a wearable patch antenna tuned to 28 GHz. (**a**) Duke model, (**b**) Ella model, and (**c**) flat phantom. For Duke and Ella, two models were considered: ‘Enriched’ (i.e., skin modelled as two layers made of the SC and viable epidermis and dermis) and ‘Original’ (i.e., skin modelled as a single layer made of the viable epidermis and dermis). For the flat phantom, two models were considered: homogeneous (i.e., skin modelled as a single layer made of the viable epidermis and dermis) and four-layer (i.e., skin modelled as two layers made of the SC and viable epidermis and dermis). For the human models, the wearable patch antenna was placed on the trunk, centred on the heart of each model.

Figure 4 shows, for each model (i.e., Duke, Ella and flat models), the psAPD averaged over 1 cm^2^ normalized to the psAPD of the SC (i.e., psAPD_SC_), considering that it is the most external layer in the Enriched models. The source in this case is the wearable patch antenna positioned at the level of the trunk.

Firstly, by comparing the results across the human models, a common trend can be observed: the exposure levels obtained when using the homogeneous version of the human model (i.e., Original) are always lower than the levels in the stratified version (i.e., Enriched), for both the viable epidermis and dermis, and the SC. More specifically, the psAPD in the viable epidermis and dermis of the Duke Original was 16.5% lower than that estimated in the SC of its Enriched version. The same was observed in the female model, Ella, where the Original version exhibited a psAPD 30% lower than that of the Enriched version. The same trend was also found with the flat models, although the difference in the psAPD between the two model versions was lower than for the human models, being equal to 11%.

Similarly, the psAPD in the viable epidermis and dermis was lower in the Original versions of the two human models than in the Enriched ones, and in the homogeneous version of the flat model vs. the four-layer version. Furthermore, focusing on each single panel of Figure 4: From panel (a) it is clear that Duke Original exhibits a lower psAPD in the viable epidermis and dermis when compared to Duke Enriched, and this difference is about 15%. The same observations can be made in panel (b), where the psAPD calculated for Ella Original in the viable epidermis and dermis was lower by 28% compared to when the same metric was estimated in Ella Enriched. Panel (c) reports the same trend in terms of a difference in the psAPD estimated in the viable epidermis and dermis in both versions of the studied models, including for the flat model: the exposure levels of the flat four-layer model are higher than the ones for the unique layer of the homogeneous model, and in this scenario this difference is about 11%.

**Figure 5:**
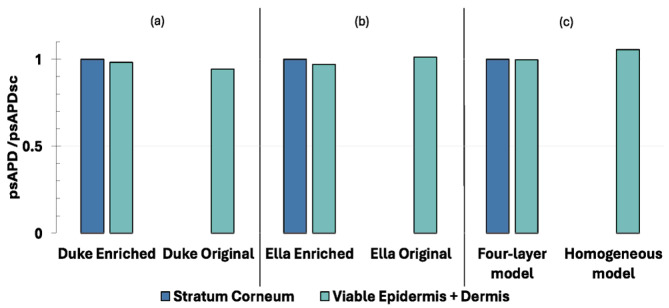
The normalized peaks of the APD averaged over 1 cm^2^ of skin tissues when the source is a plane wave tuned to 28 GHz with (**a**) the Duke model in both versions, (**b**) the Ella model in both versions, and (**c**) the flat phantom in both versions. For the human models, the region of the body exposed to the TEM-plane wave was the trunk.

**Figure 6:**
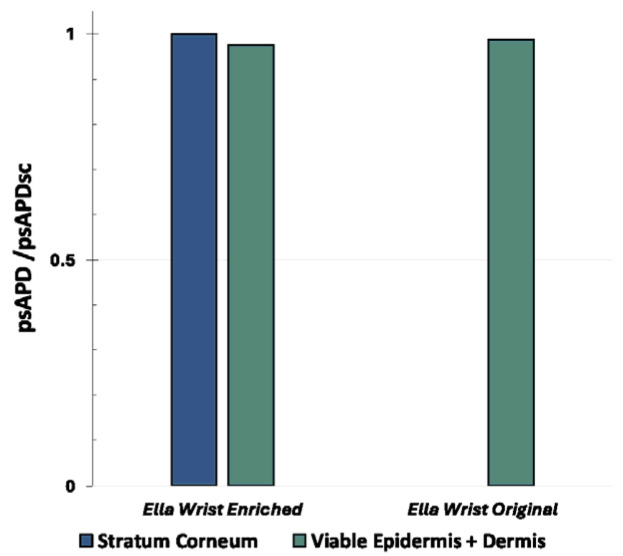
The normalized peaks of the APD averaged over 1 cm^2^ of skin tissue when the source is a plane wave tuned to 28 GHz and impinging perpendicularly on the wrist of the Ella model, both original and enriched versions.

Figure 5 shows the same type of data but obtained with the TEM-polarized plane wave. For the human models, the region of the body exposed to the plane wave was the trunk, as it was for the patch antenna. In the case of the plane wave, the differences in the psAPD values between the Enriched or Original versions of the human models and the flat model vs. the four-layer version (trunk exposure) were only marginal, of the order of 3–6%, revealing therefore the almost negligible impact of the skin model when plane wave exposure is considered. Indeed, the psAPD values in the SC of the stratified model (i.e., the Enriched one or the four-layer flat model) are almost the ones calculated in the viable epidermis and dermis of the homogenous human/flat model, with a maximum difference of about 6% in the case of Duke. The same trend can be observed in Figure 6, where the psAPDs estimated in the case of the wrist are reported.

The APD was here reported also in terms of its spatial distributions both in the scenarios where the EMF is emitted by the wearable antenna and by the plane wave impinging perpendicularly at the trunk region. Table 3 and Table 4 report the percentages of the APD values greater than 90% of the psAPD, the amount of data between 70% and 90% of the psAPD, and the percentages of data below 70% of the psAPD, for both the human and flat skin-stratified models, i.e., the Enriched and the four-layer models, in order to compare versions with the same level of detail. Examining the data in Table 3, at least the 97% of the data are below 70% of the psAPD for both the human and flat skin-stratified models. This is mainly related to the strong non-uniformity of the radiation pattern of the antenna, as expected. Moreover, in this scenario, the percentages of values > 90% of the peaks are always less than 1%, and the those of values between 70% and 90% of the peaks are at a maximum of 2.5%, revealing that these distributions are narrowed around their peak values. This trend is slightly more evident in Duke Enriched and in the flat four-layer model.

The same quantities were calculated in the scenarios with the plane wave and are collected in Table 4. Here the percentage of values exceeding 90% of the peak in the flat model represents more than 80% of the data, indicating that the distribution of the APD in the flat phantom is almost homogeneous around its peak value. The reasons are the planarity of the phantom and the uniformity of the source in terms of incidence and amplitude in the analyzed domain, and this is consistent with the opposite trend found in the case of the wearable antenna, where the radiation pattern is confined to a limited region. Concerning Duke and Ella Enriched, Table 4 clearly shows that most of the data are between 70% and 90% of the psAPD, indicating therefore that the data are more tightly gathered around the high values of the distributions but not at the peak. Moreover, Ella Enriched exhibits a higher number of values exceeding 90% of its psAPD (i.e., 35.3%), compared to Duke Enriched (i.e., 23.8%).

## 4. Discussion

Assessing human exposure to RF EMF from wearable devices is crucial given their direct contact with the body, particularly for 5G mmWave technology used in advanced wearable antennas. At these high frequencies, skin becomes the primary tissue affecting RF power absorption as the outermost organ encountering EMF. Accurate skin modelling is therefore essential for dose estimation. Two approaches exist for exposure assessment: experimental and numerical. The experimental approach uses tissue-equivalent phantoms that reproduce geometric, dielectric, and mechanical properties [26], offering significant advantages for characterizing tissue–EMF interactions [27]. However, this method has resolution limitations—current skin phantoms achieve minimum thicknesses of approximately 1 mm, which are insufficient for modelling the skin’s stratified sub-millimetric structures. Given these constraints, this work focuses on the numerical approach, which enables the implementation of very thin structures like the stratum corneum (SC). Specifically, we analyze how the absorbed power density (APD) varies with different skin modelling approaches. While the literature suggests that both two-layer skin modelling and anthropomorphic volume shape impact exposure levels, these factors have not been simultaneously investigated. Addressing this gap represents the core challenge of this study. In the present study, several scenarios were investigated, comprising the following:

(1) Two adult human models (Duke and Ella) and a flat human model, where the skin was modelled, for the first time, both as a two-layer and a homogeneous structure;
(2) Two sources of RF EMF—a realistic wearable patch antenna and a TEM-polarized plane wave with perpendicular incidence—both operated at 28 GHz;
(3) Two human body regions were exposed to the TEM-polarized plane wave to mimic different uses of wearable devices.

Overall, regarding the exposure to the wearable antenna, the psAPD estimated in the two human models was lower for the Original version (without the SC) with respect to the Enriched version. The same was observed in the flat phantoms, and this trend is consistent with the findings of Gallucci et al. [15] according to whom the variations between the APD in several multi-layer models and the homogeneous one range from 8 to 12%. Nevertheless, the effect of the skin model is evident to a lesser extent in the flat phantom than in the anthropomorphic models, and this might indicate that the actual shape of the human body has a higher impact on APD estimation. This impact of the morphology of the model on the exposure levels is in line with the results of Colella et al. [17] who estimated differences in terms of electric field induced in morphologically different models with homogeneous skin up to 2 dB; defined as not negligible.

Among the two human models, Ella exhibited the highest difference in the APD between the Original and Enriched versions, most probably because of the different shape of the chest, which is more irregular in the female model than in the male model, due to the presence of the woman’s breast. In order to quantify the irregularity of the model, the geometric excursion in the models employed was calculated as the difference between the maximum and the minimum geometric coordinates of the SC layer in the transverse plane. This difference for the Ella model is 0.15, and for Duke it is 0.11, revealing that the geometric leap is greater for Ella than for Duke; therefore, the female model is slightly more irregular than the male one. Furthermore, analyzing the data in Table 3, containing the percentages of values of the APD distribution greater or less than two thresholds, i.e., 70% and 90% of the psAPD, it is clear that these distributions are very narrowed around their peak values, regardless of the nature of the model, since the radiation pattern that affects the distribution of APD values involves a very small portion of the irradiated human body.

Regarding plane wave exposure, the psAPDs of both versions of each model (Duke, Ella, and the flat models) exhibited a high degree of similarity. This finding is consistent with the existing literature, which indicates that the presence of a thin stratum corneum (SC) (i.e., between 0.01 and 0.02 mm) has a small impact on peak exposure levels under plane wave exposure conditions [6,11]. However, these studies predominantly focus on far-field exposure, as they assume a plane wave as the source. Expanding on this evidence, the present study provides evidence that when the human body is modelled with an anatomically realistic shape and exposed to a near-field source, such as a wearable antenna, even a thin SC layer can influence exposure levels.

In order to assess the exposure in terms of the distribution of exposure metrics, the spatial distributions of the APD in the most superficial tissue of each enhanced model were studied. The data in Table 4 clearly show that the flat four-layer model reported the highest value of homogeneity, with most of the data being greater than 90% of the psAPD. Comparing the data obtained from the actual human bodies enhanced by the stratum corneum layer, Duke Enriched presented a higher level of homogeneity with respect to Ella Enriched, with 76% of the data falling between 70% and 90% of its own psAPD. Moreover, the analysis of APD distributions under the plane wave exposure conditions revealed again the influence of anatomical shape. Specifically, the female model was the one with the least uniform APD distribution. This could be due to the presence of the breast, which leads to the greater geometric irregularity of the model. This evidence is in line with the paper by Dolciotti et al. [28]: with a plane wave at 2.45 GHz, the Ella modelled with a more realistic breast in terms of volume and shape revealed a greater electric field values than in the original one.

To summarize, the comparison of the results obtained when the source was a wearable antenna and when it was a plane wave evidenced that exposure assessments obtained with a TEM plane wave are always lower than those obtained with a wearable antenna, and this is consistent with the literature [15] where this discrepancy is motivated by the fact that the plane wave is a simplification with respect to the complex radiation pattern of an antenna, and the effect of the backscattering contributing to power absorption by the biological tissue being absent when the source is a plane wave.

## 5. Conclusions

The present work aimed to study the impact of skin models on the assessment of RF EMF exposure induced by smart connected devices operated in the mmWave band. This was because there is no common consensus either in international radiation protection regulations or in the literature on the skin model to be used, and this is fundamental for EMC research areas. In this regard, two human numerical models of the ViP family, an adult female and an adult male, and a flat phantom were considered in two different versions: an Original version where the skin was modelled as a homogeneous layer made of a viable epidermis and dermis, and an Enriched version where the SC layer was added to the original model. All these models were evaluated at 28 GHz by considering two different sources of RF EMF: (i) a wearable patch antenna, and (ii) a TEM-polarized plane wave perpendicularly incident to the human models, one at a time.

To the best of our knowledge, this work is the first attempt to integrate the stratified structure of the skin and the anthropomorphic shape of human beings into a dosimetric analyses, trying to overcome the limit imposed by the typically used planar human phantom on one side and the homogeneous skin model on the other. The APD calculated through FDTD numerical simulations was always lower when using the Original version of the models for which the skin was modelled as a single homogeneous layer with respect to the Enriched versions. This trend is more noticeable in the female model and for wearable antennas. When considering plane wave exposure, the use of the Original or the Enriched version provided almost the same APD. This finding suggests that even the use of realistic human models with homogeneous skin in exposure assessment studies with wearable antennas tuned to high frequencies may underestimate the exposure.

## Figures and Tables

**Table 1:** The dielectric properties of the two layers of the multi-layered skin and the homogeneous skin at 28 GHz.

	ε_r_	σ [S/m]	ρ [kg/m^3^]
Stratum Corneum (SC)	3.52	1.21	1500
Viable Epidermis and Dermis	16	27.5	1109
Homogeneous Skin	16.5	25.8	1109

**Table 2:** A summary of the characteristics of the studies available in the literature.

	Frequency Range	EMF Source	Human Model		Parameter of Interest
			Geometry	Multi-Layer Skin	
Christ et al., 2020 [6]	6–100 GHz	Plane Wave	Flat	YES	Temperature Increase
Christ et al., 2020 [11]	6–300 GHz	Plane Wave	Flat	YES	Power transmission coefficient
Alekseev et al., 2007 [12]	37–74 GHz	Plane Wave	Flat	YES	Power reflection coefficient
Alekseev et al., 2008 [13]	30–300 GHz	Plane Wave	Flat	YES	Reflection, power density, penetration depth, SAR
Sasaki et al., 2017 [14]	10 GHz–1 THz	Plane Wave	Flat	NO	ΔT
Gallucci et al., 2024 [15]	28 GHz and 39 GHz	Wearable Antennas/Plane Wave	Flat	YES	psAPD
Colella et al., 2023 [17]	24–28 GHz	Plane Wave	Anthropomorphic	NO	Electric field
Sacco et al., 2022 [19]	26 GHz and 60 GHz	Plane Wave	Curve	NO	APD
Kapetanović et al., 2023 [20]	26 GHz and 60 GHz	Plane Wave	Anthropomorphic	NO	APD
Diao et al., 2020 [21]	6–60 GHz	Plane Wave	Curve	NO	APD and temperature increase
Present Work	28 GHz	Wearable Antenna/Plane Wave	Anthropomorphic	YES	APD, psAPD

**Table 3:** Percentages of data greater/lower than 90% and 70% of the psAPD with a wearable antenna.

	Duke Enriched	Ella Enriched	Flat Four-Layer Phantom
Data > 90% of psAPD	0.3	0.6	0.4
70% of psAPD < Data < 90% of psAPD	1.2	2.5	1.6
Data< 70% of psAPD	98.5	96.9	98

**Table 4:** Percentages of data greater/lower than 90% and 70% of the psAPD with a TEM-polarized plane wave.

	Duke Enriched	Ella Enriched	Flat Four-Layer Phantom
Data > 90% of psAPD	23.8	35.3	84.1
70% of psAPD < Data < 90% of psAPD	76	64.2	15.6
Data< 70% of psAPD	0.2	0.5	0.3

## Data Availability

No new data were created or analyzed in this study. Data sharing is not applicable to this article.
